# Implementation of a referral and expert advice call Center for Maternal and Newborn Care in the resource constrained health system context of the Greater Accra region of Ghana

**DOI:** 10.1186/s12884-020-03534-2

**Published:** 2021-01-13

**Authors:** Ebenezer Oduro-Mensah, Irene Akua Agyepong, Edith Frimpong, Marjolein Zweekhorst, Linda Amarkai Vanotoo

**Affiliations:** 1 Ghana Health Service, La General Hospital, PMB, La, Greater Accra Region, Accra, Ghana; 2 Ghana Health Service, Research and Development Division, Dodowa Health Research Center, P.O. Box DD1, Dodowa, Ghana; 3 Athena Institute, Vrije University of Amsterdam, Amsterdam, Netherlands; 4 Ghana Health Service, Greater Accra Regional Health Directorate, Accra, Ghana

**Keywords:** Implementation, Maternal health, Newborn health, Call center, Referral, Expert advice, Health systems

## Abstract

**Background:**

Referral and clinical decision-making support are important for reducing delays in reaching and receiving appropriate and quality care. This paper presents analysis of the use of a pilot referral and decision making support call center for mothers and newborns in the Greater Accra region of Ghana, and challenges encountered in implementing such an intervention.

**Methods:**

We analyzed longitudinal time series data from routine records of the call center over the first 33 months of its operation in Excel.

**Results:**

During the first seventeen months of operation, the Information Communication Technology (ICT) platform was provided by the private telecommunication network MTN. The focus of the referral system was on maternal and newborn care. In this first phase, a total of 372 calls were handled by the center. 93% of the calls were requests for referral assistance (87% obstetric and 6% neonatal). The most frequent clinical reasons for maternal referral were prolonged labor (25%), hypertensive diseases in pregnancy (17%) and post-partum hemorrhage (7%). Birth asphyxia (58%) was the most common reason for neonatal referral. Inadequate bed space in referral facilities resulted in only 81% of referrals securing beds. The national ambulance service was able to handle only 61% of the requests for assistance with transportation because of its resource challenges. Resources could only be mobilized for the recurrent cost of running the center for 12 h (8.00 pm – 8.00 am) daily. During the second phase of the intervention we switched the use of the ICT platform to a free government platform operated by the National Security. In the next sixteen-month period when the focus was expanded to include all clinical cases, 390 calls were received with 51% being for medical emergency referrals and 30% for obstetrics and gynaecology emergencies. Request for bed space was honoured in 69% of cases.

**Conclusions:**

The call center is a potentially useful and viable M-Health intervention to support referral and clinical decision making in the LMIC context of this study. However, health systems challenges such inadequacy of human resources, unavailability of referral beds, poor health infrastructure, lack of recurrent financing and emergency transportation need to be addressed for optimal functioning.

**Supplementary Information:**

The online version contains supplementary material available at 10.1186/s12884-020-03534-2.

## Background

Preventable Maternal and newborn morbidity and mortality are continuing challenges in low- and middle-income countries (LMIC); and Ghana like many of other countries in sub-Saharan Africa, was not able to attain Millennium Development Goal (MDG) 4 (reduce by two-thirds, between 1990 and 2015, the under-five mortality rate) and MDG 5 (reduce by three quarters between 1990 and 2015 the maternal mortality ratio) targets. Despite major drops in Under-five mortality failures in neonatal mortality decline drove in large part the failure to attain the MDG 4 target [1]. Similarly in relation to MDG 5, despite a fall in the maternal mortality ratio (MMR) from an estimated 740 maternal deaths per 100,000 live births in 1990 to 451 per 100,000 live births in 2008 [2], the rate of decline was and remained too slow over the era of the MDG. The averages moreover masked wide within country variation. The 2010 population and housing census estimated Ghana’s MMR at 485 deaths per 100,000 live births with wide variation between regions from 355 in the Greater Accra region to 802 in the Upper East region [3].

Over two decades ago, Thaddeus and Maine after an exhaustive review of literature identified that delays in identifying and reaching the appropriate facility (type II delays) and the delays in receiving of quality care once the woman reaches the facility (type III delays) were major contributing causes of maternal morbidity and mortality in low and middle income countries [4]. The available evidence suggests that these delays are a continuing problem in Ghana. Although the government of Ghana introduced a policy exempting pregnant women and their newborns from paying for delivery care across the country in 2005, maternal and newborn mortality continued to remain high. Studies before and after the introduction of the policy identified the quality of care in health facilities, including provider decision making on management choices and poor referral practices in health facilities as a major contributor to the poor maternal and newborn outcomes [5, 6].

In 2011, the Regional Health Directorate (RHD) of the Greater Accra Region undertook a formative study in three districts in the Region to better understand the factors influencing decision making of frontline providers of maternal and newborn care, and what interventions were required to support their decision making. The study found that apart from tacit knowledge and pre-existing knowledge providers sometimes consulted written protocols and guidelines depending on availability; and also informally used peer and expert consultation through face to face, phone calls, text messages and sometimes email. The choice depended on the urgency, the communication media available and staff familiarity with using the method. Health system constraints such as availability of staff, essential medicines, supplies and equipment as well as management issues and interpersonal relationships between staff and barriers to referral also influenced the quality of clinical decision making and potential related outcomes [7].

Among the interventions that frontline providers of maternal and newborn care felt would greatly facilitate and improve the quality and outcomes of their decision making was ready access to senior colleagues and experts for advice and support when managing obstetric and newborn emergencies, as well as an improved and coordinated referral process. As a follow up to this formative study, and working closely with the frontline providers of maternal and newborn health services in the Greater Accra region, a call center to support peer and expert consultation and referrals was designed by the Regional Health Directorate, with support from experts from the National ambulance Service and emergency clinicians.

The objectives for the design and implementation of the call center intervention were to improve maternal and neonatal health outcomes in the Greater Accra region by: (1) Facilitating the referral processes for maternal and newborn emergencies primarily (2) Providing reliable expert advice to frontline healthcare providers, (3) Identifying and assuring sustainable and affordable local financing for the recurrent maintenance costs of the intervention.

The call center was subsequently set up in partnership with a local private telecommunication network (MTN). The selection of the MTN as the local private telecommunication network to work with was based on evaluation of responses to an open call for proposals and quotations for support to develop a call center to support peer and expert consultation and coordinate referrals and expert advice in the region. An ethnographic study of the organizational functioning of the call center has been published elsewhere and provides detail on how and why the center functioned including factors such as how staff were selected and remunerated, resources and working conditions etc. [1].

The objective of this complementary paper is to present the analysis of routine process indicators of how the call center facilitated (or not) referral for maternal and newborn emergencies and provision of expert advice to frontline health providers. In the process we also present some of the challenges of intervention development and implementation, innovation and research in a resource constrained context and the effects on the call centre intervention and its functioning. This paper focuses on a formative rather than a summative evaluation of the call Center intervention to generate information on “how” and “why” the intervention works (or not) that can help to interpret any outcomes in a summative evaluation.

## Methods

### Establishment and description of the call center intervention

The call center was established with local resources contributed by the hospitals and clinics in the region from their internally generated funds (IGF). IGF comprises client out of pocket payments and National Health Insurance scheme reimbursements. Health facilities within the Ghana Health Service in the Greater Accra region agreed to contributed a portion of their IGF, based on the size of the facility. The hospitals and clinics had been part of the design of the intervention; and saw it as a response to a felt need. The fact that the agenda setting was led by and the implementation of the formative research and design of the intervention were done as a close collaborative effort with the Ghana Health Service regional, district and hospital managers as well as frontline workers in the region enabled this strong sense of ownership. The amounts were agreed on by consensus taking into account their IGF income and were the Ghana cedi equivalent of USD 600 for hospitals, USD460 for polyclinics. The smaller facilities (health centres) and small clinics with less revenue did not contribute. Ten (10) hospitals and six (6) polyclinics contributed approximately USD 15,000. This was many times less than the budget estimates that were made calculated as needed to run the intervention full scale 24 h daily and conduct formative as well as summative evaluation. The IGF contributions were paid into a pooled fund coordinated by the Greater Accra Regional Health Directorate (RHD).

Space for the housing of the center, and the costs of shared overheads such as electricity and water was provided by the Ridge Regional Hospital Out-Patients Department (OPD), Adabraka (now Adabraka Polyclinic). The Free University of Amsterdam Athena Center, as part of a research collaboration contributed through the University of Ghana’s School of Public Health, an amount of Nine thousand nine hundred and fifty Euro (€9950.00) to support the initial monitoring and documentation of the center and its effects and outputs. Beyond this there was no dedicated funding for the establishment and recurrent costs of the intervention or its monitoring and evaluation. The need to manage within these severe resource constraints was an important factor affecting how and why the intervention was implemented.

A series of stakeholder meetings were held with heads of referring (smaller hospitals and polyclinics) and receiving (larger hospitals, including secondary and tertiary) facilities to discuss and refine the modalities for the setup and functioning of the center and make sure they were responsive to the felt needs of the providers. MTN Ghana, a private telecom operator, set up the equipment to provide the Telecommunication backbone for the center between February and March 2015. The call center started full operations on 12th April 2015. The service was advertised only to the frontline health workers who refer and the receiving hospitals who take the referrals through these stakeholder engagements with facilities and their managers. These staff were regarded as the direct potential population being served by the call center or the primary users. All frontline health workers within the various health facilities within Greater Accra region (over 11 hospital, 11 polyclinics, 16 health centers, over 30 private hospitals etc.) were all part of the target population for this intervention as well as the referral hospitals receiving patients from all lower level hospitals within the region. The clients who were referred or received referral services through the center were regarded as the indirect potential population to be served by the center. This is because these clients themselves were not the ones who took the decision to use the center or who contacted the call center for service once the decision was taken. No specific advertising was targeted at clients therefore. However, our ultimate goal was to improve the health and wellbeing of the clients.

#### Functional setup and operations of the center

The Regional Call Center at inception was staffed by 8 officers who worked on a shift system. They were assisted by an administrative assistant, and supervised by a coordinator. The 8 officers comprised 3 midwives, 3 general nurses and 2 national service persons. They were trained on how to operate the center using the Ghana Safe Motherhood protocols over a two-day period in March 2015 by the Chief Nursing Office, the Deputy Director of Nursing in charge of Clinical Care and Emergencies, the Call Center Coordinator, staff of the Institutional Care Division of the Ghana Health Service and the National Ambulance Service. These officers were responsible for carrying out the technical functions of the call center i.e. coordinating access to expert support and referral coordination. The center operated under the office of the Regional Director of Health Services. The coordinator had oversight responsibility for the day-to-day running of the facility. The center primarily supported maternal and neonatal units of all health facilities within the region for two main functions of the coordination of expert advice and referrals.

#### Coordinating expert advice

**Fig. 1:**
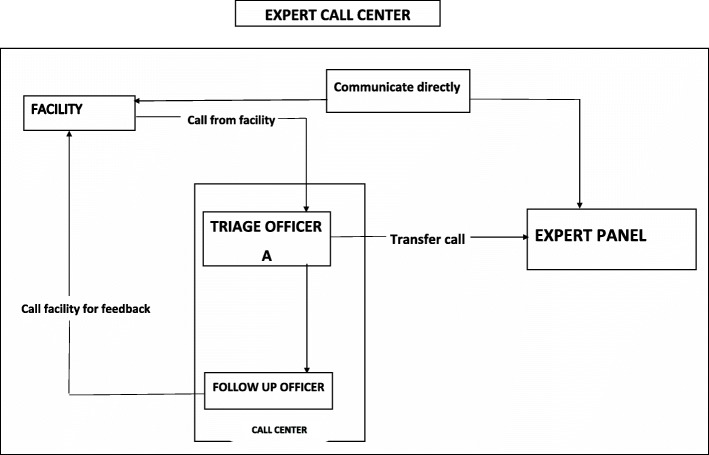
Flow chart showing the direction of communication between the call center, lower level facilities requesting expert advice and the expert panel

To coordinate expert advice, the center worked with an expert panel of senior practitioners to aid frontline maternal and newborn health (MNH) service providers at all levels of care in the region. Frontline service providers were able to reach the experts by a phone call routed through the center. The expert panel had a membership of three obstetricians, two paediatricians, the regional pharmacist and an anaesthetist. These officers were employees of the Ghana Health Service and did not receive any special remmuneration for providing advice. It was treated as part of their normal work. The center officers received calls for help directly from frontline health service providers, and immediately transfered the call to the appropriate expert panel member based on the nature of the request. Follow up officers were as part of the standard operating procedure (SOP) required to call health facilities back within 15 min of transferring their calls to the expert panel members for feedback. Where a referral was required, either by information from the expert panel or feedback from the health facility, the follow-up officers proceeded to arrange for the referral. This mechanism is illustrated in Fig. 1.

#### Coordinating referrals of emergencies

The call center coordinated referrals between health facilities, based on bed availability and the availability of personnel to manage the patient’s condition. Staff of the Call Center worked closely with the National Ambulance Service (NAS) and the major referral hospitals in Accra (Ridge Regional Hospital, Tema General Hospital, LEKMA Hospital, La General Hospital, Achimota Hospital, Pentecost Hospital, Korle-Bu Teaching Hospital and 37 Military Hospital) to ensure that referrals were handled efficiently.

**Fig. 2:**
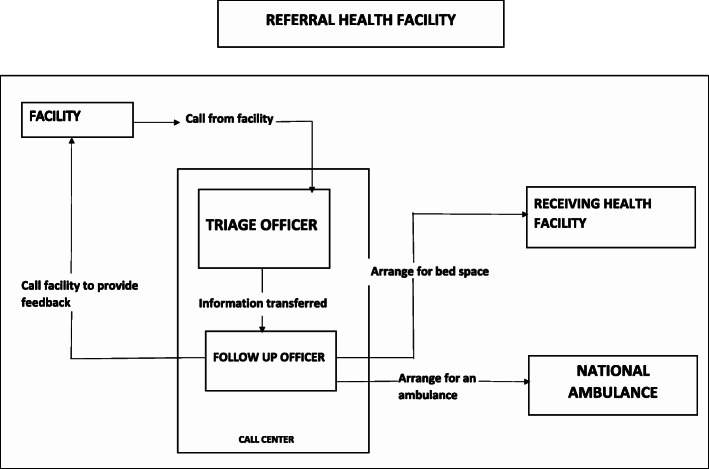
Flow chart showing the direction of communication between the call center, referring facilities, receiving facilities and the national ambulance service

When a frontline worker needed to refer an emergency, a call was placed by the frontline worker to the call center. The officer at the center answered the call and conducted a brief telephone interview of the caller to document the details of the call on the Call Center Documentation form, and then transferred the call to the follow-up officer. It was the responsibility of the follow-up officer to ensure that a bed was secured in a health facility and an ambulance was dispatched where possible to transport the patient to the receiving facility (Fig. 2). This was to ensure that effective inter-facility referral communications were made before referrals with the main aim of ensuring that receiving facilities were pre-informed and were ready to receive the patients.

In all cases, it was Standard Operating Procedure (SOP) for any missed phone call to be returned within 15 min. Call logs on the phones were routinely checked to verify this. It is however possible that a few calls may have been missed and therefore, not documented.

#### Phases of operation of the call center

Phases of operation of the call center can be split into the first 17 months (phase 1) when a private telecommunications company, MTN, providing the ICT platform (April 2015 to August 2016) and the period after the exit of MTN (phase 2) when the platform relied on the limited but free Gota phone system provided by National Security.

##### Phase one: April 2015 – august 2016 (17 months)

The center operated with a backbone support for the Information Communication Technology (ICT) equipment provided by MTN Ghana. Operations started with 6 triage officers (3 midwives, 3 general nurses) and two national service personnel. The staff operated from 8 pm to 8 am i.e. run 12-h night shifts, with at least one midwife and one general nurse on duty per shift. Calls expected to be received at the center were to be for either expert advice or referral purposes. The running of 24-h services in phase 1 was constrained by inadequate resources for the recurrent operational costs. The original concept and design was for 24 h but faced with these resource constraints, priority was given to operating the center between 8 pm to 8 am, when the need for expert or specialist support for frontline health workers was greatest. The extra resources that were not immediately available to make a 24-h service possible were mainly for recurrent costs such as phone bills, stationery, additional staff, staff training etc. The challenge occurred because health facilities that had initially pledged to make financial contributions to support the center reneged on their pledges as they themselves struggled with inadequate financing. Ghana has moved to lower middle-income status and many development partners that supported the health sector are transitioning out. Unfortunately, resources available for service delivery at the frontline from government taxes are also declining.

Apart from the startup costs already described, health facilities had agreed and were required to make recurrent monthly contributions to the regional health directorate to help pay for the cost of maintaining the contract with MTN Ghana. As these payments continued not to be made as agreed, the service provide MTN Ghana could not be paid and they eventually disconnected the phone lines of the center on 31st August 2016. This challenge was addressed when the National level took an interest in the center’s activities, and provided GOTA phones and additional staffing to man the center in September 2016.

##### Phase two: September 2016 – December 2017 (16 months)

With the discontinuation by MTN of its support to the center because it was unable to meet the recurrent cost, the center from September 2016 operated with GOTA phones which are dedicated security phones donated by the National Security Service through the Ghana Health Service. Cordless and mobile GOTA phones were also distributed across most health facilities. In all, twelve facilities received cordless referral Gota phones. This was made up of nine (9) Ghana Health facilities, two (2) quasi-government hospitals and one (1) teaching hospital. A phone was also given to the National Ambulance Service. All health facilities who received Gota phones were referral level hospitals i.e. received emergencies from lower level health facilities.

These advantage of these phones and the service was that they were provided free by government. The disadvantage was that the scope of service available under this system was less than under the commercial system run by MTN and they could not be used for conference calling, call transfers and expert advice. Hence, the role of the call center in providing expert advice was curtailed. The center could now only support referrals between facilities.

The introduction of the Gota phones, and provision of extra staff for the call center by government through the Regional Health Directorate allowed a decision to run the center for 24 h instead of 12 h, and to open the center to receive calls for all clinical emergencies, not just maternal and newborn care. Additional personnel were trained and brought on board, making a total of 10 officers (5 clinical staff and 5 non-clinical staff, with the sixth clinical staff taking up other duties in another unit). These officers run 12 h shifts i.e. day and night duties; with at least two on duty in a given shift.

### Post phase 1 and 2

The call center has continued to operate after this 33-month pilot period, and has been upgraded to a teleconsultation center by the Ghana Health Service.

### Study design, data collection methods and analysis

#### Study design

The study design was a longitudinal time series routine data analysis of process indicators of the immediate outcomes of call center functioning over a 33-month period starting from April 2015 when the centre commenced operation. This covers the 17 months of phase 1 and an almost equivalent period of 16 months of phase 2.

#### Data collection methods and analysis

At inception of the operation of the center, a simple form (Supplementary 1) was designed for the call center staff to manually fill as they responded to calls. The staff documented on this form, each call, made to the call center, the source clinic or hospital (including caller and phone number), referral diagnoses, cases received at first attempt, those received at subsequent attempts, outcomes of referrals and expert advice. The advice algorithm used by the staff at the call centre to guide their decision making on each call is attached as Tables 1 & 2.

**Table 1:**
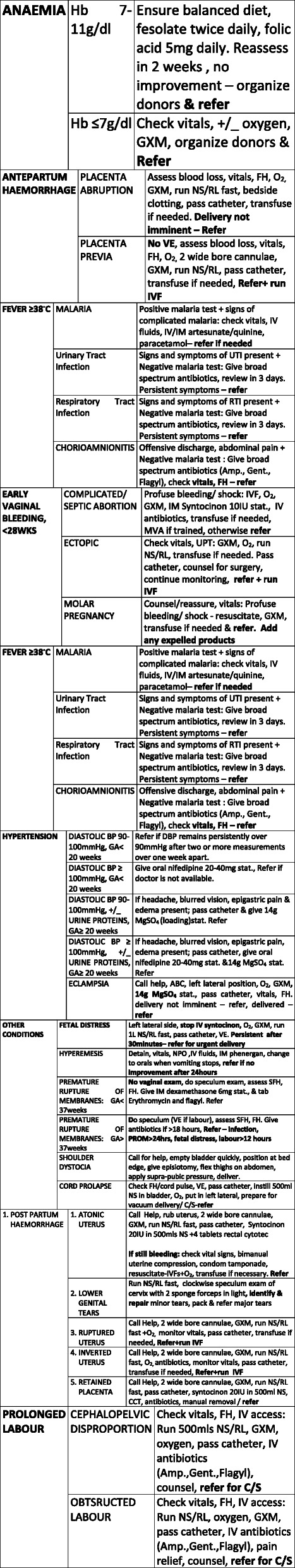
Maternal protocol for call center

**Table 2:**
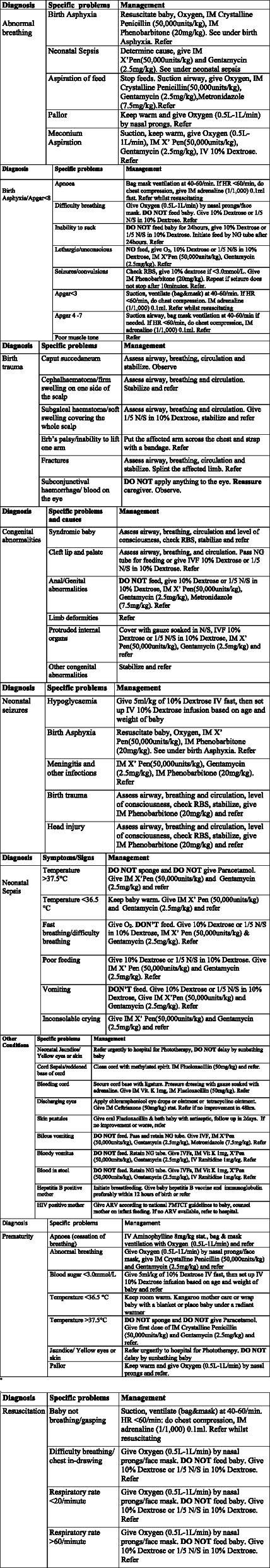
Newborn protocol for call center

Some receiving facilities failed to respond when their emergency lines were called on some days, or had their phones off, making it difficult for the call center to reach them. Some staff members were delegated as focal persons for the call center in their health facilities. Their role was to coordinate activities of the call center within their facilities, providing regular updates on the bed states of their facilities and serving as a point of contact if the call center is facing difficulties accessing a bed. However, follow-up officers were not able to perform this task as no phones were provided for them specifically for this role due to the financial constraints under which the intervention was implemented. The data generated was regularly checked for completeness and consistency and then entered into Excel. We analyzed this data in Excel for frequencies and trends.

## Results

### Phase one: MTN as the ICT platform handler (April 2015 – august 2016)

A total of 372 calls were handled by the call center between 15th April 2015 and 30th August 2016 when the ICT platform was handled by the private telco, MTN. Just about 2.4% of these calls were requests for expert advice (1.9% for obstetrics and 0.5% for neonatal conditions), while 93% of calls received were requests for referrals (Table 3).

**Table 3:** Types of requests to the call center between April 2015 and August 2016

Type of request	No.	Percentages
Referral - Obst.	322	86.6
Referral - Neonatal	24	6.4
Referral - Medical	16	4.3
Expert - Obst.	7	1.9
Expert - Neonatal	1	0.5
Ambulance request only	2	0.3

Most calls were recorded between the hours of 9 pm in the evening and 12midnight and between 6 am to 7:30 am. With regards to request for referrals, about 86.6% (322 out of 362 calls) were requests for obstetric referrals and a few were for neonatal referrals (24 cases). Though designed specifically for maternal and newborn care, the center was not completely restricted to these cases. It also received a few requests (16 calls total, 4.3% of all calls) to support the referral of general medical and surgical emergencies such as severe anemia and severe hypertension. There were two (2) requests for ambulance support.

#### Reasons for referral

Referring facilities gave several reasons why they needed to refer their patients. The health system factor underlying most referrals was the unavailability of skilled personnel to handle the complications of labour and delivery at the point of referral. The clinical reasons for obstetric referral are presented in Table 4, with the most common reasons being prolonged labour, and pregnancy induced hypertension and its complications and postpartum haemorrhage. The most common clinical reasons for newborn referral were birth asphyxia (58.3%) and prematurity (16.7%).

**Table 4:** Reasons for obstetric referrals, showing frequency of occurrence in percentages

Reason for obstetric referral	No.	Percentage
Prolonged labour	81	25.1
PIH and Complications	56	17.4
PPH	21	6.5
APH	19	5.9
Abnormal presentation	18	5.6
Foetal distress	18	5.6
Delayed 2nd stage	17	5.3
Prev. C/S	15	4.7
CPD	9	2.8
Big abdomen	9	2.8
Meconium stained liquor	7	2.2
PROM	6	1.9
Retained placenta	5	1.5
Twin gestation	5	1.5
Others	36	11.2

#### Response by referral receiving facilities

**Fig. 3:**
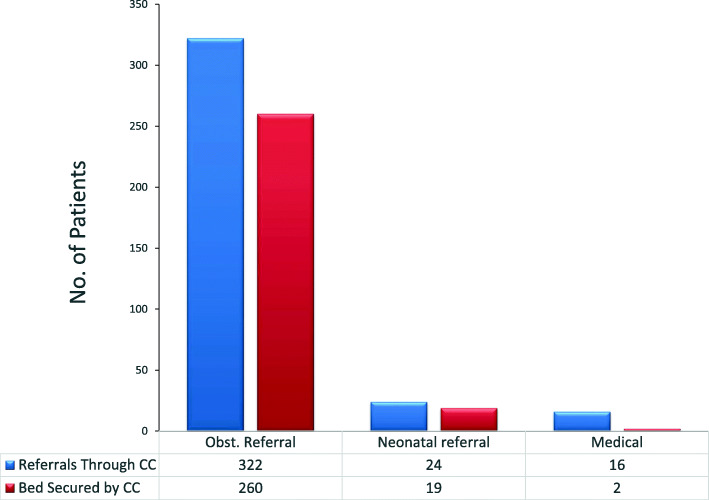
Number of referrals and beds served

The overall response rate by receiving facilities was very good. Of 322 obstetric requests for bed space at a receiving facility, 260 beds were secured (Fig. 3), giving a success rate of 80.7%.

Almost all referrals were to hospitals with the majority (75%) going to the Greater Accra Regional Hospital, Ridge. (Table 5).

**Table 5:** Patients received by referral facilities during phase 1

Receiving facilities	No. received	Percentage
Ridge Regional Hospital (GARH)	211	75
La General Hospital (LGH)	22	7.8
37 Military Hospital	19	6.8
Korle Bu Teaching Hospital (KBTH)	16	5.7
LEKMA Hosp.	5	1.8
Achimota Hospital	2	0.7
Pentecost Hospital	1	0.4
Not indicated	5	1.8
	281	100

Hence, the overall total referral success rate over the period was 77.6, and 22.4% of patients that needed referral could not be referred because there was no bed available in almost all the major referral facilities called. Sometimes, when the call center was unsuccessful in securing a bed for the client and the case was critical; staff members were still told to send their patients to the receiving facility while a solution was negotiated to save critical time. On arrival, the call center staff communicated directly with the staff at the receiving facility to admit the patient.

**Fig. 4:**
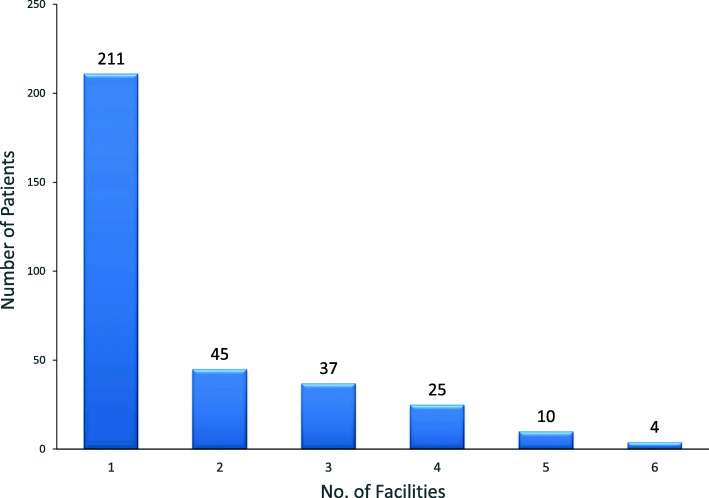
Number of facilities called before securing bed space

The call center could reach receiving facilities at the first attempt in 211 cases representing 63.6% of cases. In a small number of cases (1.2%), it took up to six attempts to reach a referral facility (Fig. 4).

#### National Ambulance Services

The call center received a lot of support from the National Ambulance Services (NAS). The NAS was able to provide an ambulance for 85 patients out of the 139 requests for ambulance services (61.2% success rate). For the nearly 39% of patients that the NAS was not able to serve, reasons given by the NAS and documented by the call center personnel included lack of fuel, the ambulance on other assignments or the ambulance undergoing servicing. Some of the health facilities made arrangements for their own ambulance, and therefore did not need the services of the call center to reach the NAS.

### Phase two: post MTN (September 2016 – December 2017)

#### Types of request

Due to the withdrawal of services by the telecommunication company MTN, only the referral platform was operational from September 2016. A total of 390 calls were made to the center from September 2016 to December 2017. All the calls were for referral purposes, made up of medical emergencies (50.5%), obstetrics and gynaecological emergencies (30.3%), paediatric emergencies (8.7%), surgical emergencies (6.7%) and requests for ambulance services (3.8%).

#### Facilities calling and successes

**Fig. 5:**
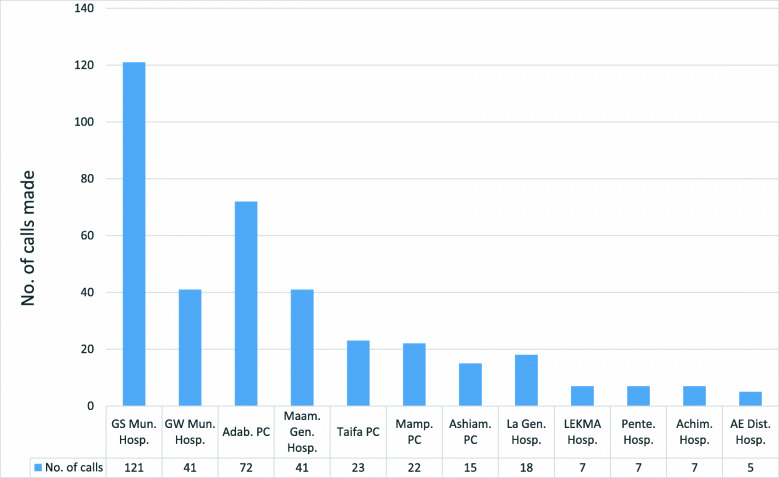
List of facilities who called the call center for support between September 2016 and December 2017

Most calls came from primary level facilities (district hospitals and polyclinics) to other primary level facilities, secondary or tertiary facilities with the required expertise to manage the cases. (Fig. 5). Of the 92 requests for ambulance services, 71 were served, giving a success rate of 77.2%. It must be emphasized that the call center run side-by-side with the referral whatsApp platform operated and coordinated by another organization.

### Response by referral receiving facilities

**Fig. 6:**
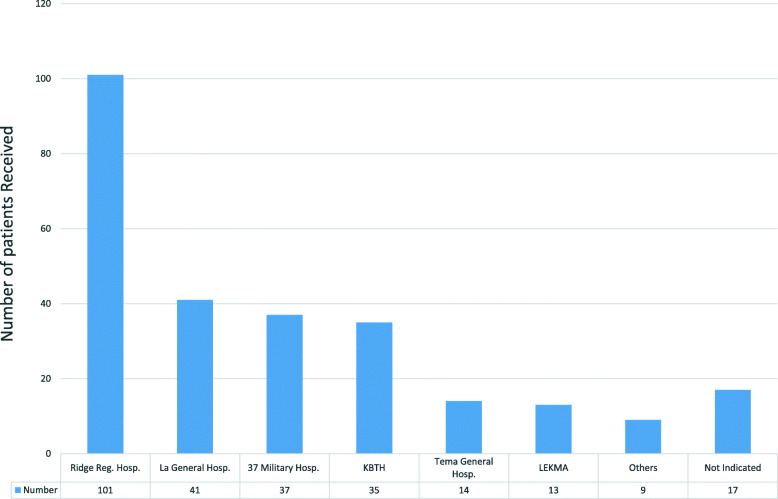
Number of referred patients received by each referral facility between September 2016 and December 2017

There were 390 requests for beds of which 267 (68.5%) were honoured. There was no indication of whether patient was received or not for six referrals. One patient died before arrangement for referral could be completed. Of the 267 patients served with a bed through the call center, receiving facility was not indicated against 16 of them. Of the remaining 250 patients, as in phase 1, Ridge Regional Hospital received 40.4% of the case forming the majority (Fig. 6). About 31.5% of referrals could not get a bed (receiving facility) because all the referral facilities contacted by the center indicated their beds were full.

**Fig. 7:**
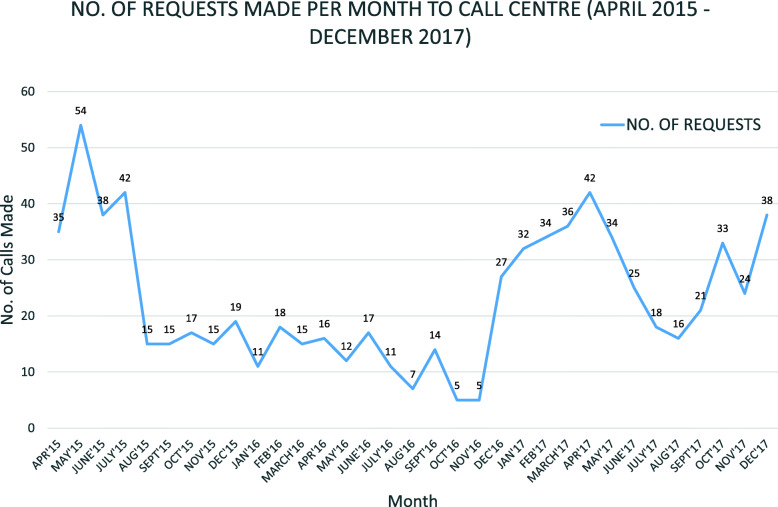
Number of calls from health facilities to the call center per month between April 2015 and December 2017

Over the 33-month period that the data was analyzed, the total number of calls per month ranged from 5 to 54, with an average number of 23 calls per month. Much of the low average was driven by a severe drop in the number of calls per month between the months of August 2015 and November 2016 (Fig. 7).

## Discussion

This ultimate aim of the call center intervention whose process or intermediate outcomes are analyzed and presented in this paper is to reduce referral delays and also to improve quality of care for referred mothers and newborns. Delays in identifying and reaching the appropriate facility and delays in receiving of quality care once the woman reaches the facility remain major contributing causes of maternal morbidity and mortality in low and middle income countries since they were described over two decades ago [4]. The call center intervention was designed to help address delays in decision making as well as identifying a health facility for patients needed emergency specialist care and transferring them promptly. This was by the call center helping to identify quickly a facility with the required expertise ready to receive the emergency, and facilitating the transport of the patient to that facility. The difficulties in this area in Ghana and the tragic consequences have attracted wide spread attention from the public and parliament and has acquired the name “no bed syndrome” (e.g. https://www.graphic.com.gh/features/opinion/no-bed-syndrome-a-telling-phenomenon-of-ghana-s-health-care.html; https://www.gbcghana.com/1.11658719. Accessed 18–6-20) to describe the situation of referred patients moving from one health facility to another in a private vehicle, taxi or ambulance, looking for a facility with the required expertise and/or bed availability to manage their condition. The call center intervention was also designed in part to address delay in receiving quality care on reaching a health facility. Health facilities agreeing to receive an emergency referral where pre-informed through the call center system, and so better prepared to receive patients and address emergency needs. This reduced instances where emergencies had to queue to be attended to because facilities were unprepared, in terms of human resource and logistics to receive those emergencies.

Referral systems and Clinical decision-making support systems are potentially important interventions for improving maternal and newborn health outcomes by reducing these types of delays. The current implementation research study has focused on developing, implementing and monitoring the operational effects and feasibility of an intervention that tries to improve the situation using a call center to provide expert advice and coordinate referrals. Additionally, the research has explored how to implement such an intervention with locally available resources in a resource constrained setting. Beyond the core research questions of analyzing the process indicators of call center functioning the fact that this intervention and its monitoring and evaluation have been done almost entirely with such funding as could be mobilized locally and very little to no external funding is in itself learning about the challenges of research and innovation in a resource constrained context. Despite the resource challenges that never completely resolved the work was carried with modification and improvisation as a response to the resource constraints out because the intervention was a strong felt need in context. There can be incredible complexities involved in setting up and monitoring what may appear ‘a simple intervention’ in a low resourced country or context. This paper is therefore as much about the call center as it is about the challenges of research and innovation in an LMIC context with limited government and almost no donor funding. This includes adapting intervention design and monitoring and evaluation methods to the resources available rather than giving up because the desired ideal is not possible. The lessons from the intervention and its monitoring and evaluation as well as carrying out research and innovation under resource constraints are of relevance not only to Ghana but to other low- and middle-income countries.

The findings of study suggest that the call center intervention that has been developed in the Greater Accra Region with the frontline health workers and managers and in response to their felt needs is doable and worth continued piloting, monitoring and evaluation to document its impact is on maternal and newborn health outcomes. The study has also clearly shown that there are several health system and contextual challenges that need to be addressed concurrently to effectively scale up and sustain the intervention and document its impact on maternal and newborn health outcomes in the medium to long term.

Firstly, there was the challenge of the limited number of receiving facilities and inadequate beds in relation to need that caused delays in referrals. This “No bed” syndrome is a long standing problem in the health sector in the Greater Accra region [7–9]. .The region has the fastest population growth in the country due to migration in and its infrastructure lags behind the population needs. The call center helped to coordinate referrals and potentially minimized referral delays but could not completely eliminate them partly because of this challenge. The “no bed” syndrome was responsible for majority of unsuccessful referrals. Providing adequate referral infrastructural capacity and human resource for maternal and neonatal health referrals is an urgent need that has to be tackled if the unfinished MDG 4 and 5 agenda which has now become part of the SDG are not to remain unfinished.

Secondly even where a referral hospital had beds available there was sometimes a problem of a lack of skilled staff in the night to attend to emergencies because of the challenge of inadequate human resource numbers as well as skill mix in the region. Despite the coping strategies put in place by facilities [10, 11], the problem persists because there is an absolute shortage. Since patient loads are lower at night, one such coping strategy is that skilled staff are preferentially put on the heavy day shifts. However, it means that most health facilities in the GAR do not have the required skilled personnel in the night needed to manage some of the maternal and newborn health complications that turn up then, even when there are beds to admit the patient. Some of the referrals made by the referring facilities could have been avoided if frontline maternal and neonatal service providers were better skilled. Capacity development programs for emergency obstetric and neonatal care service providers especially at the primary health facilities could reduce the burden on any call center and also on referral beds.

Thirdly there was sometimes a lack of the needed equipment, tools and supplies as well as finances for the referral facilities to perform optimally. Some staff who had been dedicated as focal persons by their facilities to provide regular updates on bed state for maternal and newborn care in their facilities did not have dedicated phones for this activity. Effectively they had to use their own phones and pay for the cost of any calls. Thus, providing regular updates on bed state to the call center presented a huge challenge for them. Also, the call center services provided by the expert panel members were additional task to their regular work, meaning extra hours of work. However, given the funding constraints it was not possible to provide any remuneration for overtime as recognition of this added duty.

Fourthly and possibly related to the demotivation provided by some of the challenges outlined above, sometimes the response and support from some of the referral facilities was poor. Sometimes, calls to referral facilities were not answered or phones were turned off at the receiving end. Also, in some cases the referring facilities placed a call for help through the follow-up officers’ phone number instead of the triage phone number. This potentially delayed response times by making the routing more circuitous.

The low call volumes recorded during the latter part of 2015 was in part related to the onset of a nation-wide medical doctors’ strike in July 2015, which lasted through to September 2015. Even after the strike ended, the underutilization pattern continued through till December 2016 when the center started operating 24 h. Another factor that probably accounted for some of the reduction in calls to the center even after the doctors strike ended was the introduction of other interventions by other partners in health development. Specifically, there was the introduction of a WhatsApp-based platform to facilitate referrals and expert advice for frontline health workers dedicated to maternal health services in early 2016. Although it covered just 50% of facilities, its ease of access meant most frontline personnel enrolled onto this platform. Thus, a number of maternal and newborn related cases which would have been routed through the call center was now managed over the whatsApp based platform, significantly reducing call loads to the call center. This however demonstrated the negative impact of multiple independent unintegrated initiatives during the implementation and evaluation of impact of new interventions.

The private telecommunication company providing the IT platform in the first 17 months of operation of the Center had a Customer Care and Monitoring Software that tracked where calls are coming from, who was receiving the calls, time call came in among other important information. However, this software was not free and the Call Center was unable to raise enough money to purchase this tool. All the documentation, which was essential for monitoring and evaluation, therefore had to be manual. This introduced extra workload for the staff.

A major challenge the call center faced was finding sustainable ways to finance the recurrent costs of the call center such as the operative and maintenance cost, and the cost of running regular stakeholder meetings on the call center. This work has shown that it is possible to mobilize funds locally for an intervention of this nature. However, the resource constrained context remains a challenge that limits how much can be mobilized and requires constant creativity and adaptation. This constraint sometimes meant the call center had to manage in a less than optimal way. Incentives such as overtime allowances that were supposed to be provided for the expert panel members for the additional hours they spent to provide services to patients if the intervention is to be sustained on a long term basis, could not be provided. Similarly a simple need to provide phones for the follow-up officers to improve their functioning could not be met. Countries like Ghana which are seen as transitioning or recently transitioned from being low income to lower middle income and needing less development assistance can be particularly challenged by not being “poor enough” and yet not “rich enough”.

The problem with referral transportation and staff to accompany referrals is another major challenge. It is not a new one. Nkyerkyer in a study of peripartum referrals to the Korle-bu teaching hospital documented that as much as 72% of the referrals travelled to the hospital by public or private transport and 54% were not accompanied by any staff [5]. In a more recent study Atuoye et al. [12] also document the referral transportation challenge. Awoonor-Williams et al. [13] reported a study of pre post intervention audits of five (5) emergency obstetric and neonatal referral networks in the Upper East region of Ghana. Multiple micro-interventions selected by the facilities in the program included how to complete and use the partograph, posting and hiring new staff and drivers, infrastructure improvement, provision of additional equipment and supplies (including cell phones) and more intensive engagement with drivers and emergency transport preparation. Whether the referred woman was accompanied (and by whom) affected how quickly the referral got executed, the cost of referral and how stable her condition was upon arrival. There were also issues with whether the National Ambulance Service was available or referrals transportation had to be by taxi and motorbike. The availability of an ambulance was sometimes limited by the fact that the ambulances on duty were inadequate or may have been assigned to other emergencies, and so were not immediately available to assist the center.

In literature search to inform this study, we found very little on call center interventions to facilitate clinical decision making and referral for mothers and newborn in LMIC; but found several initiatives to improve communication between health facilities and emergency transportation with the aim of reducing transfer delays of both obstetric and neonatal cases in low and middle income countries. For example, in Southern Malawi, a repeater- based VHF communication system was installed in health centres for direct communication with district health offices to arrange for referral transport (Lunga and Ratsma, [14]). In India, the implementation of the JSY cash programme to enhance access to EmOC facilities, produced some unintended effects such as adverse birth outcomes (Chaturvedi et al. [15]). This problem was felt to be likely to have been caused by low quality of referral services and dearth of other amenities at the EmOC and EmNC facilities such as basic supplies, drugs, equipment, and the inadequate number of surgery facilities as found in other settings (Cavallaro and Marchant, [16]; Fikree et al. [17]). In rural Burundi, a joint EmOC facility and car ambulance service was associated with an increased coverage of complicated obstetric cases and caesarean sections resulting in reduced maternal and new-born deaths (Tayler-Smith et al. [18]). In Malawi and Zambia, the use of emergency transport systems such as motorcycle ambulances, bicycle ambulances, boats, oxen and donkey carts, depending on the terrain, caused a significant reduction in referral delays (Surridge et al. [19]; Bhopal et al. [20]; Hofman et al. [21]) coupled with high community acceptability, accessibility and value for ambulance services (Surridge et al. [19]; Bhopal et al. [20]). Similar findings of enhanced access to, and the use of maternal and new-born health services; increased institutional deliveries; reduced maternal deaths due to the availability of free-of-charge 24-h ambulance services have been found in different settings including Uganda, Honduras, Sri Lanka, Sierra Leone, Malaysia, Nepal, South Africa (Prinja et al. [22]; Mucunguzi et al. [23]; Schoon, [24]; Murray and Pearson, [25]; Samai and Sengeh, [26]).

It will be important to link emergency transportation access to any call centre intervention for support decision making and facilitate and reduce referral delays in LMIC. The findings of challenges with emergency transportation in this study despite the fact that the Greater Accra Region is better served than the rest of the country, confirms this need for that a multi-team as well as multi-sectorial approach that includes interventions to improve emergency transportation and road access. Similar observations have been made in other LMIC e.g. [Essien e. Ifenne D., Sabitu K., Musa A., et al. 1997 International Journal of Gynecology and Obstetrics 59 supplement 2 S237-S244. Community Loan Funds and transport services for obstetric emergencies in Northern Nigeria].

This study was conducted in the Greater Accra Region of Ghana, which is 90% urban and relatively better served than much of the country. It is therefore open to the critique that perhaps the circumstances of the study setting are very different from the rest of Ghana and much of rural sub-Saharan Africa. However, the limited literature we found on referral interventions and experiences in Ghana suggest that the challenges and needs in other parts of Ghana may have much in common with the study setting. Afari et al. 2014 [6] in a qualitative study to understand local healthcare worker perspectives on cause of challenges in the referral of obstetric cases in Assin North, a rural district in the Eastern region of Ghana found that contextual factors such as roads and road security as well as transportation limited referral systems. There were also challenges with inadequate care (stabilizing and monitoring the patient) during transportation between referral and receiving facility. Part of it was related to the use of non-ambulance transport that is not suitably equipped for emergency obstetric care. There were also communication barriers related to the fact that there were no standard systems for communication on emergency obstetric care so any communication is dependent on the initiative of the individual provider e.g. give personal phone numbers to patients, call ahead to receiving units, accompany patients. Related to the communication problems, lack of advance notice from referring health centers makes it more difficult for hospital staff to prepare adequately. Inadequate staff numbers and skills were sometimes also a problem e.g. no staff to accompany referred patients in critical condition, late recognition of danger signs and taking of appropriate action.

Additionally, despite the perception of its seeming “privilege”, the Greater Accra region is particularly challenged by the massive migration into the region and heavy urbanization (now over 90% urban) without corresponding increase in infrastructure, equipment, tools and supplies and human resources for health. As rural urban drifts continue and urbanization increases across Africa, attention needs to be paid to the potential challenges of urban health in low resource settings.

Although the call center was set up purposely to reduce the need for referrals, the low volumes of requests for expert advice (2.4% in phase 1) bring to the fore the lack of skilled personnel, lack of resources or lack of both in lower level facilities. This suggests the need for staffing, capacity building and resourcing of health facilities to manage for complicated medical conditions at lower levels of care. This is because most frontline workers considered the need to refer patients higher and above the need to seek for expert advice to manage patients in their facilities.

### Limitations of the study

The design and piloting of the call center intervention within the real world constraints of limited resources and with a bottom up participatory approach has provided valuable insights about implementing a referral and decision making support call center despite the resources constraints of a low and middle income country. We know how and why the intervention works (or not) in context. We have however not evaluated its effectiveness in terms of its impact on maternal and newborn health outcomes. That will be important to do as the next stage of this effort. The lack of an electronic call recording, documentation and storage system prevented us from identifying calls which may have been missed due to absence of center officers. This may have affected the validation of actual total number of calls received.

## Conclusion

A call center to coordinate referrals and expert advice is a potential viable E-Health intervention to support improved Maternal and Newborn care, as well as other clinical emergencies in an LMIC. However, to achieve its full benefit, it needs to be implemented whilst concurrently addressing health systems challenges such staffing, referral beds, emergency transportation and road access. It has been possible to mobilize some domestic resources to support the intervention. Advocacy and resources for maternal and new born survival needs to be tied to advocacy and resources for health systems strengthening. However adequate financing without external support remains a challenge in the resource constrained context of an LMIC. In a country like Ghana which is now classified as a lower middle income country and is in the process of transitioning to “Ghana beyond AID”, it is critical to invest more attention to issues of how to make support research to help identify financially sustainable, potentially lifesaving innovations and interventions.

## Supplementary Information

**Additional file 1: Supplementary 1.** GAR/GHS Emergency Referral and Coordination Centre

## Data Availability

The study received approval from the Regional Health Directorate, who are the owners of both the call center and data generated from the call center. The data that supports the findings of this study is available from the corresponding author with the permission of the Greater Accra Regional Director of Health Services, Ghana upon reasonable request. The call center is overseen by the Greater Accra Regional Health Directorate, and the data generated is part of routine documentation at the call center on a day to day basis. Being data from services provided to clients and health facilities, they are not publicly available.

## Abbreviations

IGF: Internally Generated Funds
LMIC: Low- and Middle-Income Country
MDGs: Millennium Development Goals
MNH: Maternal and Newborn Health
NAS: National Ambulance service
RHD: Regional Health Directorate.

## Footnotes

1 Frimpong E., Oduro-Mensah E., Vanotoo L. & Agyepong I.A. (2018). An exploratory study of the organizational functioning of a decision making and referral support call center for frontline providers of maternal and newborn care in the Greater Accra region of Ghana. International Journal of Health Planning and Management 2018; 1–12
