# Supplementary material for: Implementation of a referral and expert advice call Center for Maternal and Newborn Care in the resource constrained health system context of the Greater Accra region of Ghana
Source: BMC Pregnancy Childbirth. 2021 Jan 13;21:56. doi: 10.1186/s12884-020-03534-2 (PMC7807452; doi:10.1186/s12884-020-03534-2)
Supplement: Supplementary file 1 — Additional file 1: Supplementary 1. GAR/GHS Emergency Referral and Coordination Centre [file 12884_2020_3534_MOESM1_ESM.pdf]

GAR/GHS EMERGENCY REFERRAL AND COORDINATION CENTRE

Date(dd/mm/yy):	Time of call: (24hr)	Serial No.:
Health Facility Calling:	District:	
Name of staff calling:	Cadre:	
Phone number:		

**Request Type**		
Referral:<br><br>a) Obstetrics / Gynaecology<br>b) Paediatrics<br>c) Medical<br>d) Surgical	Expert Advice:<br><br>a) Obstetrics / Gynaecology<br>b) Paediatrics<br>c) Medical<br>d) Surgical	Other Requests:
Reason for request:		
If referral, ambulance needed?	a) Yes	b) No

IMMEDIATE FOLLOW-UP FEEDBACK:

**Receiving referral facility:**<br>Contact at facility: Time call placed:<br>Time National Ambulance Service called: Time bed secured:			
**Feedback to facility initiating call**			
Facility<br>1. Time call placed:<br>2. Contact at facility:	Bed secured<br>1. Yes<br>2. No	Ambulance secured<br>1. Yes<br>2. No(reason)	Expert Advice<br>1. Expert contacted<br>2. Patient referred

LATE FOLLOW-UP FEEDBACK

**Feedback from facility initiating call**<br>State of patient on arrival<br>1. Alive 2. Alive but morbid 3. Dead 4. Unknown<br>Comments:
**Comment by Triage Officer on referral process:**
